# Ultrasound-Guided Moving Shot Radiofrequency Ablation of Benign Soft Tissue Neoplasm

**DOI:** 10.3390/medicina57080830

**Published:** 2021-08-17

**Authors:** Wei-Che Lin, Yi-Fan Tai, Meng-Hsiang Chen, Sheng-Dean Luo, Faye Huang, Wei-Chih Chen, Pi-Ling Chiang, Hsiu-Ling Chen, Mei-Hsiu Chen, Jung-Hwan Baek

**Affiliations:** 1 Department of Diagnostic Radiology, Kaohsiung Chang Gung Memorial Hospital and Chang Gung University College of Medicine, Kaohsiung 833, Taiwan; eggivon@hotmail.com (Y.-F.T.); sperfect1101@gmail.com (M.-H.C.); lovage@cgmh.org.tw (P.-L.C.); suring.tw@gmail.com (H.-L.C.); 2 Department of Otolaryngology, Kaohsiung Chang Gung Memorial Hospital and Chang Gung University College of Medicine, Kaohsiung 833, Taiwan; rsd0323@cgmh.org.tw (S.-D.L.); jarva@cgmh.org.tw (W.-C.C.); 3 Plastic and Reconstruction Surgery, Kaohsiung Chang Gung Memorial Hospital and Chang Gung University College of Medicine, Kaohsiung 833, Taiwan; spencerfaye@hotmail.com; 4 Department of Beauty Science, Meiho University, Pingtung 912, Taiwan; x00003116@meiho.edu.tw; 5 Department of Radiology and Research Institute of Radiology, University of Ulsan College of Medicine, Asan Medical Center, Seoul 05500, Korea; radbaek@naver.com

**Keywords:** radiofrequency ablation, benign soft tissue neoplasm, cosmetic issue, volume reduction, anesthesia

## Abstract

*Background and Objective*: To evaluate the effectiveness of radiofrequency ablation (RFA) using the moving-shot technique for benign soft tissue neoplasm. *Materials and Methods*: This retrospective study reviewed eight patients with benign soft tissue neoplasm presenting with cosmetic concerns and/or symptomatic issues who refused surgery. Six patients had vascular malformation, including four with venous malformation and two with congenital hemangioma. The other two patients had neurofibroma. All patients underwent RFA using the moving-shot technique. Imaging and clinical follow-up were performed in all patients. Follow-up image modalities included ultrasound (US), computed tomography (CT), and magnetic resonance (MR) imaging. The volume reduction ratio (VRR), cosmetic scale (CS), and complications were evaluated. *Results*: Among the seven patients having received single-stage RFA, there were significant volume reductions between baseline (33.3 ± 21.2 cm^3^), midterm follow-up (5.1 ± 3.8 cm^3^, *p* = 0.020), and final follow-up (3.6 ± 1.4 cm^3^, *p* = 0.022) volumes. The VRR was 84.5 ± 9.2% at final follow-up. There were also significant improvements in the CS (from 3.71 to 1.57, *p* = 0.017). The remaining patient, in the process of a scheduled two-stage RFA, had a 33.8% VRR after the first RFA. The overall VRR among the eight patients was 77.5%. No complications or re-growth of the targeted lesions were noted during the follow-up period. Of the eight patients, two received RFA under local anesthesia, while the other six patients were under general anesthesia. *Conclusions*: RFA using the moving-shot technique is an effective, safe, and minimally invasive treatment for benign soft tissue neoplasms, achieving mass volume reduction within 6 months and significant esthetic improvement, either with local anesthesia or with general anesthesia under certain conditions.

## 1. Introduction

Cosmetic issues are among the primary problems reported in patients with benign soft tissue neoplasms, particularly those with lesions in the head and neck region. Indeed, the issue is of such importance that these patients are often compelled to seek further medical treatment. Although the majority of benign soft tissue neoplasms are asymptomatic, some of these lesions may become symptomatic and/or experience progressive growth. These bulging lesions, including vascular malformations, neurofibromas, or other lesions, commonly cause symptoms such as compression sensation, swelling, paresthesia, pain, and contour deformity [1,2,3]. In patients with lesions in the head and neck region, these gradually growing lesions may lead to perceived esthetic issues, particularly facial asymmetry [4]. Of note, interventions for facial asymmetry are recommended in the early stages of lesion growth, as correction in more advanced stages often presents further complications [5].

Managements for benign soft tissue neoplasms have been reported, including operation, sclerotherapy, and trans-arterial embolization (TAE), depending on the nature and location of the lesion [3,6]. Surgical excision is a definite treatment, but presents several obstacles [7,8], including scar formation, morphologic deformity, nerve/vascular injury, bleeding [3,9], and possible reconstructive surgery [1]. As risks of cosmetic problems indeed exist, patients aiming to resolve existing cosmetic issues often prefer a minimally invasive intervention to surgery [9,10]. Due to the abovementioned shortcomings, an effective and minimally invasive intervention treatment modality with fewer complications or even single-session management is preferable.

Ultrasound-guided radiofrequency ablation (RFA) is a minimally invasive procedure for use on benign and malignant tumors, including hepatocellular carcinoma, hepatic metastatic tumor, thyroid nodules, parathyroid adenoma, musculoskeletal soft tissue tumors, and parotid Warthin tumors [11,12,13,14,15,16]. In contrast to the conventional fixed electrode technique during ablation, RFA using the moving-shot technique facilitates real-time monitoring of the procedure, making it possible to perform RFA in more delicate locations and to avoid vital structure damage [17]. Currently, RFA using the moving-shot technique is widely applied to thyroid nodules, yielding volume reduction, cosmetic improvements [18,19], and a low complication rate [20].

To date, limited literature exists reporting on the application of RFA using the moving-shot technique in benign soft tissue neoplasms, including vascular malformation and neurofibroma. In the present study, we review and report on the safety and efficacy of RFA using the moving-shot technique in the treatment of benign soft tissue neoplasm.

## 2. Materials and Methods

### 2.1. Patient Population and Evaluation

This study was approved by the institutional review board and written informed consent was waived. The patient inclusion criteria were as follows: (1) pathology confirmation and/or image diagnosis of benign soft tissue neoplasm; (2) presence of cosmetic concerns and/or symptoms; (3) superficially located mass facilitating the ultrasound-guided procedure; (4) unwillingness to undergo surgery.

From March 2016 to July 2020, we retrospectively reviewed eight consecutive patients who had benign soft tissue neoplasm and underwent US-guided RFA treatment at a 2000-bed tertiary referral medical center in southern Taiwan. The demographic data of the patients are shown in Table 1.

Of the eight patients, six had vascular malformation, including four with venous malformation confirmed by biopsy and two with clinical and image-diagnosed congenital hemangioma. The other two patients had neurofibroma, which was also confirmed by biopsy. All patients sought medical help due to cosmetic concerns and/or symptomatic issues. They all received information regarding their respective lesions and the variety of treatment options available, including conservative follow-up, surgery, and RFA. All eight patients refused surgery.

### 2.2. RFA Technique

For each patient, the pre-operative evaluation for mass location included ultrasound (US), computed tomography (CT), and magnetic resonance (MR) imaging. All targeted venous malformations were located at the head and neck region, including the buccal space, the infratemporal fossa, and the mouth floor. One of the congenital hemangiomas was located at the lateral abdomen wall, the other at the buttock region. Both targeted neurofibromas were located at the temporal fossa. According to the pre-operative image study, all targeted lesions were superficially located.

The RFA was performed percutaneously under real-time US guidance using the moving-shot technique [17]. Two of the eight patients received the procedure under local anesthesia with administration of 2% lidocaine hydrochloride solution in an outpatient setting; the remaining six patients received the procedure under general anesthesia. An internally cooled electrode (18 gauge, with 5 mm, 7 mm, or 1 cm active tip) with RF generator (VIVA, STARmed, Gyeonggi-do, South Korea, and M2004, RF Medical, Seoul, South Korea) were used. Under the US, the targeted mass was conceptually divided into multiple compartments. The electrode tip was first inserted into the deepest compartment and the ablation was conducted. The selection of the RF energy (10–50 watts of RF power) was based on whether the emergence of an echogenic area around the electrode tip during each RF procedure. The electrode was pulled slowly to ablate the adjacent compartment, and the mass lesion was then sequentially ablated from the deepest compartment to the most superficial compartment. The endpoint of each procedure was when all compartments turned into transient hyperechoic zones. The total ablation time was <20 min in each case. All RFA procedures were executed by one author (15 years of experience in image-guided procedures).

### 2.3. Follow-Up and Outcome Measurement

Image and clinical follow-up were performed in all patients. Follow-up image modalities included US, CT, or MR imaging. Clinical information, including symptoms, cosmetic scale, and complications, was recorded at the initial evaluation and at each follow-up after RFA treatment. Follow-up within 2–6 months after RFA was defined as the midterm follow-up, and follow-up within 1–2 years after RFA was defined as the final follow-up. The mass volume was measured by CT/MR with an off-line workstation (AZE Virtual Place v3.4, Tokyo, Japan), using the same image modality for each patient. The remnant mass volume post RFA was divided by the volume prior to RFA to calculate the volume reduction ratio (VRR). All the above measurements were performed by two radiologists with 10–15 years of experience.

The cosmetic scale applied in the current study was modified from the established scale which has been used for years to evaluate results of thyroid nodules treated with minimally invasive procedures [19]. The scale has values from 1 to 4 and categorizes different cosmetic improvements as follows: 1—no visible or palpable mass; 2—no visible but palpable mass; 3—mild bulging mass lesion with vague border; and 4—obvious mass lesion with distinct border.

There were several complications of US-guided RFA that had been reported, including skin burn, scarring, surrounding nerve or vascular injury, infection, active bleeding, hematoma [10,20,21,22]. Major complications were defined as any event that required a certain level of treatment/intervention or prolonged hospitalization. All other events without sequelae or requiring no or nominal therapy were defined as minor complications [23].

### 2.4. Statistical Analysis

Statistical analyses were performed using SPSS 24 (SPSS, Inc. Chicago, IL, USA). All data were expressed as mean ± standard deviation. The statistical analysis of the cosmetic score change was performed using the Wilcoxon signed-rank test. One-way analysis of variance was used to compare the volumes according to the follow-up period for each patient. A *p*-value <0.05 was considered significant.

## 3. Results

Among the eight patients in this study, seven patients had no previous treatment. One patient (patient 3) had received venous malformation excision 15 years prior to the RFA procedure. The median follow-up period after the procedure was 12 months (range, 6–28 months). Follow-up imaging for seven patients was performed using MR, while the other patient (patient 1) had contrast-enhanced CT due to a metallic implant. The maximum diameter of each mass prior to RFA was 5.5 ± 1.4 cm (range, 3.3–7.1 cm).

**Figure 1:**
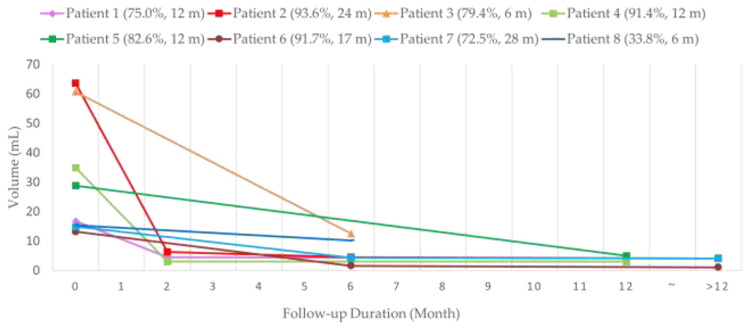
Trend Chart of Mass Volume Reduction after radiofrequency ablation (RFA). m = month(s).

**Figure 2:**
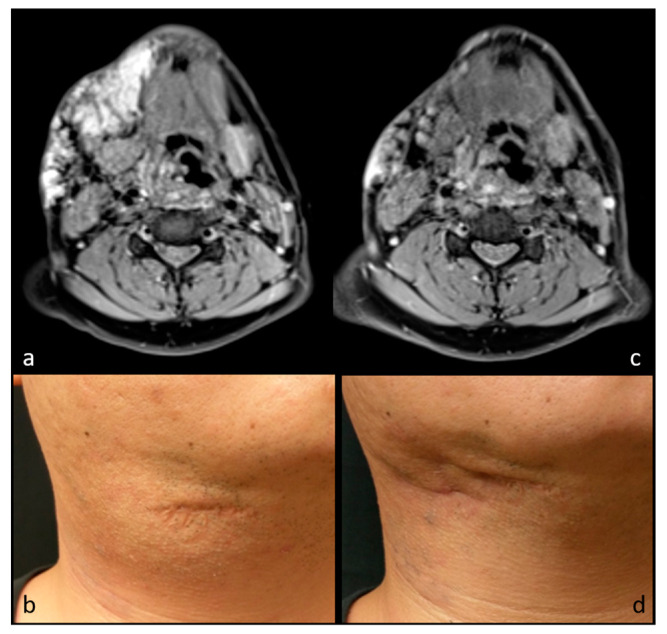
MR images and photographs of patient 3 before (**a**,**b**) and after (**c**,**d**) RFA (cosmetic scale 3 → 1). Previous surgical scar is also visible in photographs.

**Figure 3:**
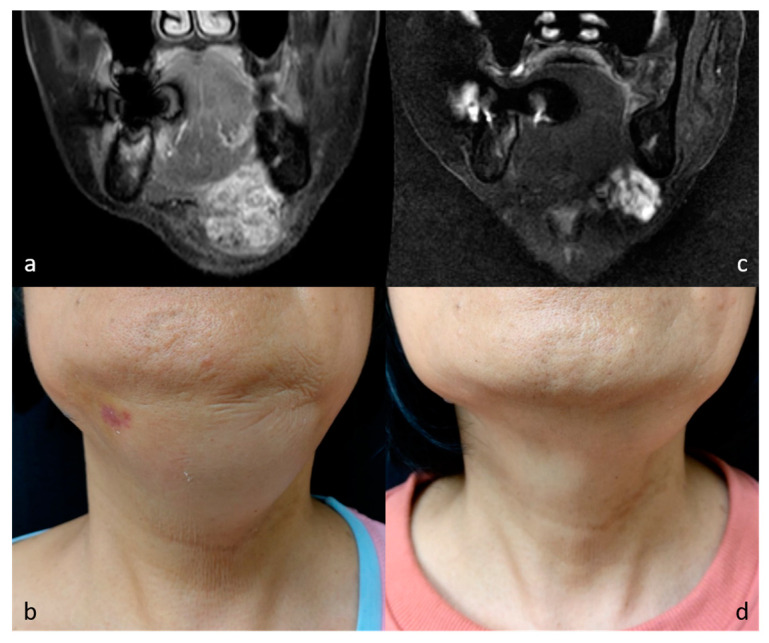
MR images and photographs of patient 4 before (**a**,**b**) and after (**c**,**d**) RFA (cosmetic scale 4 → 1).

Seven patients received single-stage RFA. The mean volumes of each mass before RFA, at midterm follow-up, and at final follow-up were 33.3 ± 21.2 cm^3^ (range, 13.2–63.6 cm^3^), 5.1 ± 3.8 cm^3^ (range, 1.6–12.5 cm^3^), and 3.6 ± 1.4 cm^3^ (range, 1.1–5.0 cm^3^). There were statistically significant volume changes between baseline volumes, midterm follow-up (*p* = 0.020), and final follow-up (*p* = 0.022) after RFA. The VRR were 82.7 ± 9.4% at midterm follow-up and 84.5 ± 9.2% at final follow-up. There was also significant volume reduction for patients with vascular malformation (41.0 ± 20.4 cm^3^ vs. 5.8 ± 3.8 cm^3^, *p* = 0.014). As for the two patients with neurofibroma, the volumes of each mass before and after RFA were 14.1 ± 1.2 cm^3^ and 2.6 ± 2.1 cm^3^, respectively. The VRR before and after RFA for vascular malformation and neurofibroma were 84.4 ± 7.9% and 82.1 ± 13.6%, respectively. A trend chart of the mass volume is shown in Figure 1. All patients presented with a bulging lesion leading to apparent esthetic issues and pressure sensation, with cosmetic scores of 3–4 at initial evaluation. Facial asymmetry was apparent for six patients with head and neck region masses, and the patient with congenital hemangioma presented with lobular, reddish bulging mass lesion. No complaints of worsening symptoms or cosmetic problems after RFA treatment were noted during the follow-up period. Of note, there were significant improvements to cosmetic scores before and after RFA (from 3.71 to 1.57, *p* = 0.017). Photographs and image comparisons are shown in Figure 2 and Figure 3. The patients were followed up for a mean period of 14.6 months (range, 6–28 months). During the follow-up period, no significant regrowth of targeted masses was observed.

The remaining patient (patient 8) was in the process of a scheduled two-stage RFA for the congenital hemangioma. The exophytic lesion in the buttock region is vulnerable to skin burn and tissue necrosis. The first RFA procedure targeted the deep portion of the mass and deliberately spared the vascular structure. The mass volumes before and 6 months after RFA were 15.4 and 10.2 cm^3^, respectively. The VRR at the time of study reporting was 33.8%. A second RFA for the remnant lesion will be arranged with further focus on the vascular structure and improved mass volume reduction. The overall VRR in all eight patients was 77.5%, demonstrating successful and effective mass reductions.

All RFA were uneventful, without peri-procedural complications. Most patients complained of mild local pain and heat around the RFA lesion, which were successfully treated with application of ice packs and acetaminophen administration within the first few days after the RFA treatment. No major or minor complications were noted during the follow-up period. The two patients who received RFA under local anesthesia in an outpatient setting were discharged within 30 min after the procedure. The six patients who received RFA under general anesthesia were admitted for anesthesia-related evaluation and care. Four of these six patients were discharged on the day following the procedure, and the other two patients were discharged between 2 and 4 days after the procedure. These patients delayed their discharge due to personal issues or the undergoing of other unrelated examinations.

## 4. Discussion

### 4.1. Summary

At 6 months after the RFA procedure, all targeted lesions had significant volume changes with a mean VRR of 82.7%. Compared with the VRR at the final follow-up (mean, 85.6%), the mass volume change at midterm follow-up showed a much more salient decrease. These results indicate the early achievement of mass volume reduction within 6 months of RFA treatment of benign soft tissue neoplasm.

### 4.2. Cosmetic Concerns

**Figure 4:**
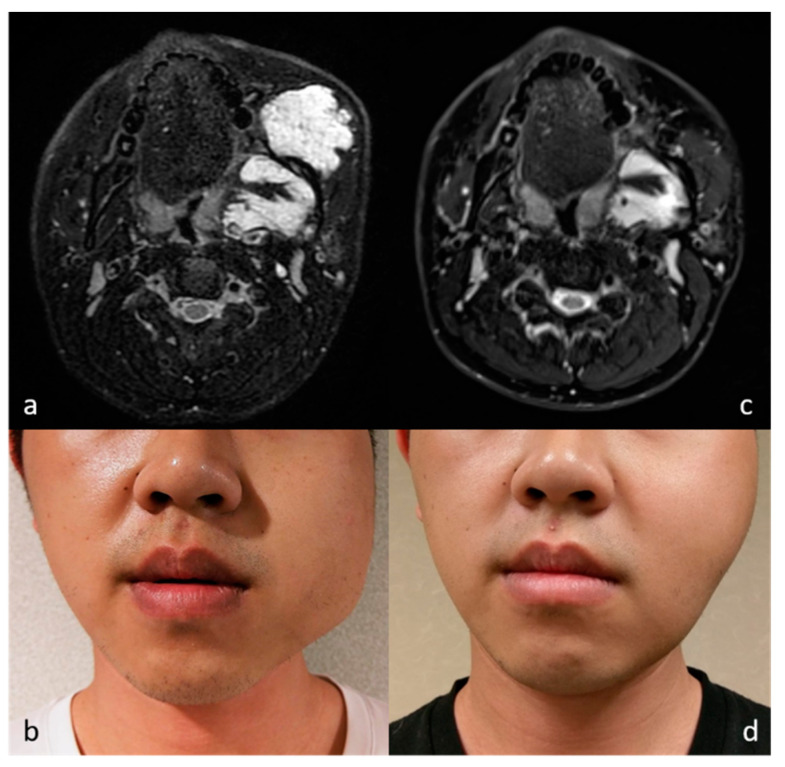
MR images of patient 2 before (**a**) and after (**c**) RFA show almost total volume reduction of the targeted venous malformation in the left masticator space. The lesion in the deep layer remained stable in size. Photographs before (**b**) and after (**d**) RFA show satisfactory improvement of facial asymmetry (cosmetic scale 4 → 3). Residual facial asymmetry was due to bone remodeling and deep-seated lesions.

**Figure 5:**
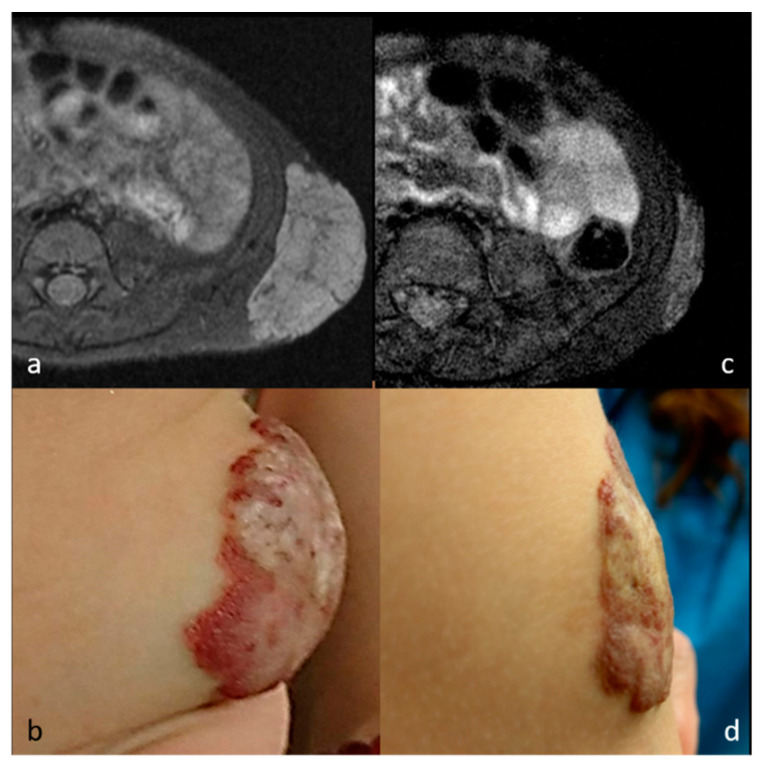
MR images of patient 5 before (**a**) and after (**c**) RFA show significant shrinkage of the congenital hemangioma in the left abdominal wall. Photographs before (**b**) and after (**d**) RFA show the cutaneous and subcutaneous lobular, reddish bulging lesion marked flattened and decolorized into pinkish color (cosmetic scale 4 → 3).

All our patients sought intervention primarily due to cosmetic concerns. Most lesions became impalpable and invisible in the follow-up period. In two patients, an almost complete volume reduction was achieved after RFA, while it must be noted that a cosmetic scale reduction to 1 is challenging to achieve. In one case (patient 2, CS: 4 → 3, Figure 4) with venous malformation in the left masticator space, while the targeted lesion shrank almost entirely, the mass lesion in the deep layer of masticator space remained stable in size during follow-up. Improved but still apparent facial asymmetry was due to bone remodeling by the long-standing lesion and deep-seated lesions beyond the reach of US-guided procedure. However, for this particular patient, the improvement in left facial bulging was satisfactory. In the other case (patient 5, CS: 4 → 3, Figure 5) with congenital hemangioma in the lateral abdominal wall, the lobular reddish bulging mass became markedly flattened and decolorized during the follow-up. Although still a visible lesion, the caregiver was also satisfied with the improvement. The results indicate that in certain patient groups, residual cosmetic problems are inevitable due to structural deformities such as bone remodeling or with pigmentation. Nevertheless, even including the above patients with residual cosmetic problems, significant cosmetic score reductions and satisfactory improvements can still be achieved. The score reductions noted herein approximate those achieved in thyroid nodules treated with RFA [18,19] and venolymphatic malformations treated with combined sclerotherapy and RFA [10]. We conclude that RFA treatment for soft tissue neoplasm can achieve significant cosmetic scale improvements and satisfactory esthetic results.

### 4.3. Vascular Malformation

With regards to vascular malformation, the mainstays of management include surgery, percutaneous sclerotherapy, and even TAE. In a recent study of surgeries in head and neck venous malformations, although excellent outcomes were realized in localized and well-defined lesions, patients with bigger and/or ill-defined lesions yielded less effective outcomes [9]. Another major consideration of surgical excision is post-operative complications affecting up to 10% of patients [1,9]. Percutaneous sclerotherapy has been replacing surgery in first-line treatment for venous malformation [24]. Several concerns over sclerotherapy include often requiring multiple treatment sessions, possible lesion re-expansion, and considerable complication rate, which may include tissue or skin necrosis, nerve damage, and pulmonary embolism [24,25,26]. In a large study, sclerotherapy for head and neck venous malformation was successful in 82% of patients, with an overall complication rate of 7.7% [27]. Recently, RFA has been applied to venous malformation in the head and neck region [28]. In another study, 10 cases of smaller venous malformation (mean, 18.6 cm^3^) in the head and neck region, treated with real-time US-guided RFA, showed a median volume reduction at 6 months of 55.7% [21]. Compared with other treatments and previous RFA studies, our study demonstrates better volume reduction without complications or scar formation. Hence, US-guided RFA using the moving-shot technique may provide an effective and safe alternative treatment.

### 4.4. Neurofibroma

Regarding neurofibroma, conservative observation may be sufficient for most asymptomatic patients, but may eventually lead to operation during follow-up [2]. Complete resection may only be possible in relatively small and confined tumors [29]. While radical resection is challenging to achieve for the majority of cases, incomplete resection independently associates with increased local recurrence [3]. Relatively limited literature has reported on the application of RFA for treatment of benign peripheral nerve sheath tumors. A pilot study reported on multiple courses of RFA in five cases of craniofacial plexiform neurofibromatosis with equivocal results [30]. Two cases of benign retroperitoneal nerve sheath tumors treated with CT-guided RFA showed significant tumor shrinkage, and symptoms were relieved without recurrence during follow-up [31,32]. To our knowledge, this is the first study to report on two cases of head and neck neurofibroma treated with RFA using the moving-shot technique. Both patients with neurofibroma received uneventful RFA treatments and demonstrated positive results without local recurrence during follow-up, further confirming the indication of RFA as an effective alternative method for relieving symptoms and cosmetic problems in patients with neurofibroma.

### 4.5. Local Anesthesia vs. General Anesthesia

Currently, there is no consensus regarding which anesthetic technique should be applied in RFA, or which patients should receive general anesthesia for soft tissue neoplasm RFA [33]. Most RFA procedures using the moving-shot technique are conducted under local anesthesia [18,34,35,36]. Typically, general anesthesia for RFA is only considered in patients with various levels of anxiety and pain [37]. However, soft tissue neoplasm can have complex surrounding anatomical structure or indistinct border under US. In the cases reported herein, general anesthesia ensured better patient compliance; furthermore, it may not only enhance operator confidence but has also become essential to accomplish thorough ablation and avoid injury of surrounding vital structures [33]. In addition, the infiltrative nature of targeted lesions may result in less distinctive borders under echography. This may lead to incomplete local anesthesia, suboptimal pain control, and it may further affect the procedure [38,39]. Of note, in pediatric patients, RFA is a feasible and promising treatment for tumors of various origins [40,41]. In younger patients, RFA under general anesthesia can guarantee immobility throughout the procedure. Our team has further extended the usage of RFA to congenital hemangioma and neurofibroma in pediatric patients, with the youngest patient having received RFA at the age of 11 months. However, it should be noted that several disadvantages of RFA under general anesthesia indeed exist, including hospitalization requirement and potential concerns, such as delayed detection of peri-procedural complications including nerve injury and skin burn [20,21,22]. Therefore, if the lesion is well-defined under US and reasonable patient cooperation is anticipated, local anesthesia is sufficient for RFA. However, when encountering indistinct lesion borders under US and/or concern of poor compliance due to anxiety, excessive pain, or pediatric patients, RFA under general anesthesia is recommended to achieve better pain control, prevent peri-procedural complications, and enhance operator confidence.

## 5. Limitations

Several limitations to this study should be addressed. First, only small number of cases were included in the study. Second, there were only a limited variety of benign soft tissue neoplasms, confined to vascular malformation and neurofibroma. Third, follow-up times in this study were variable and relatively short. Although our results demonstrated the efficacy of RFA in reducing mass size and addressing cosmetic issues, a longer follow-up period would be recommended for evaluation of potential mass regrowth. Fourth, as one case was in the process of a scheduled two-stage RFA, the results of the two-stage RFA would be important to include in a future study.

## 6. Conclusions

In conclusion, US-guided RFA using the moving-shot technique is an effective, safe, and minimally invasive treatment for benign soft tissue neoplasm exhibiting significant volume reduction and esthetic improvement. This study demonstrated early achievement of mass volume reduction within 6 months post RFA without documented complications or local recurrence in the follow-up period. For certain patients, RFA under general anesthesia is recommended if borders are indistinct under US or concerns of poor patient compliance exist.

## Figures and Tables

**Table 1:** Demographic Information.

Patient Number	Age (Year)	Sex	Diagnosis	Location	Image Modality	Volume (cm^3^)		Follow-Up (Month)	VRR ^a^	CS ^b^		Anesthesia Method	Discharge after RFA (Day)
						Pre	Post			Pre	Post		
1	27	F	Venous Malformation	Right maxillary	US, CT	16.8	4.2	12	75.0%	4	1	LA	-
2	22	M	Venous Malformation	Left masticator space	US, MR	63.6	4.1	24	93.6%	4	3	GA	1
3	30	M	Venous Malformation	Right mandible	US, MR	60.7	12	6	79.4%	3	1	LA	-
4	59	F	Venous Malformation	Left side mouth floor	US, MR	34.9	3.0	12	91.4%	4	1	GA	2
5	1	F	Congenital Hemangioma	Left abdominal wall	US, MR	28.8	5.0	12	82.6%	4	3	GA	1
6	16	F	Neurofibroma	Left temporal fossa	US, MR	13.2	1.1	17	91.7%	3	1	GA	4
7	5	F	Neurofibroma	Right temporal fossa	US, MR	14.9	4.1	28	72.5%	3	1	GA	1
8 ^c^	7	F	Congenital Hemangioma	Left buttock	US, MR	15.4	10.2	6	33.8%	4	4	GA	1

M = male; F = female; Pre = before RFA; Post = after RFA; LA = local anesthesia; GA = general anesthesia. ^a^ VRR = volume reduction ratio = 1 − (volume before RFA)/(volume after RFA). ^b^ CS = cosmetic scale: 1 = no visible or palpable mass; 2 = no visible but palpable mass; 3 = mild bulging mass lesion with vague border; 4 = obvious mass lesion with distinct border. ^c^ Patient 8: in process of a scheduled two-stage RFA.

## Data Availability

Data sharing not applicable.
